# Knowledge and experience of paramedics concerning patients with hearing and visual disability

**DOI:** 10.1186/s12873-023-00866-y

**Published:** 2023-08-17

**Authors:** Nesrin Alharthy, Raghad Almotairy, Rahaf Aldulhum, Albatool Alghamdi, Reem Aquil, Ghada Alkharaan, Sara Alsuwais, Abdullah Alshibani

**Affiliations:** 1 Pediatrics Emergency Department, King Abdullah International Medical Research Center, King Saud bin Abdulaziz University for Health Sciences, King Abdulaziz Medical City, Riyadh, Saudi Arabia; 2 Emergency Medical Services Department, College of Applied Medical Sciences, King Saud bin Abdulaziz University for Health Sciences, Riyadh, Saudi Arabia; 3 King Abdullah International Medical Research Center, Riyadh, Saudi Arabia

**Keywords:** Hearing disorders, Vision disorders, Emergency medical services, Prehospital, Communication

## Abstract

**Background:**

A 2017 nationwide disability survey conducted by the General Authority of Statistics in Saudi Arabia, a sample representing the whole population living in Saudi Arabia, reported that approximately 5% and 2% of the Saudi population suffers from visual or hearing impairments, respectively. Patients with these disabilities find it difficult to convey their medical history and chief complaints to paramedics, causing communication breakdowns that can lead to misinterpretation of patient history, leave medical problems unaddressed, and reduce patient engagement and autonomy. We aimed to assess paramedics’ knowledge, attitude, and level of confidence when managing patients with visual or hearing problems.

**Methods:**

Descriptive cross-sectional design was used to report the knowledge and experience of paramedics towards patients with hearing/vision disabilities in Saudi Arabia. A validated questionnaire was distributed to our study sample of paramedics in Riyadh, Saudi Arabia between 01, July 2020 and 31, December 2020. Ethical approval was obtained from King Abdullah International Medical Research Center.

**Results:**

Ninety-seven participants completed the survey. Male paramedics accounted for 77% of the study participants; 24% were Saudi Red Crescent employees, and 57% were 20–25 years old. Most participants encountered 1–5 cases of patients with hearing disability (55%) as well as patients with visual disability (48%) during their career. Taking medical history was a challenge indicated by 42% of the participants, and 30% reported difficulties in explaining procedures. Of the participants, 44% were confident in handling patients with hearing or visual impairment. There was a strong association between participants who indicated higher confidence levels and those who had obtained specific training for patients with hearing or visual impairments.

**Conclusion:**

Assisting patients with hearing or visual impairments is challenging, especially during an emergency. We recommend programs that provide specific training in handling hearing or visually impaired patients to close the communication gap in emergent medical situations handled by paramedics or other emergency medicine doctors and nurses.

**Supplementary Information:**

The online version contains supplementary material available at 10.1186/s12873-023-00866-y.

## Introduction

Emergency care providers including paramedics, whose purpose is to respond to any patient requiring emergency care, attend to a variety of cases. The cases they encountered in their daily practice may vary from very critical to minor cases [1]. One of the key skills that paramedics need to master is effective communication skills especially with patients. Such communication allows developing the rapport between the healthcare provider and the patient, facilitating sharing of information (e.g., chief complaint and medical history), improving compliance of patients with treatment, and optimizing overall patient satisfaction [2–4].

However, there are certain cases which could be challenging to paramedics such as responding to patients with hearing or visual disability who require emergency care. “Disability” is a broad term that covers defects in the function or structure of the body which are categorized depending on the type of defect a person may have and the respective magnitude of the impairment’s impacts on daily activities [5]. The impairment’s impact on daily activities mainly refers to functional disability, which is defined as acquired difficulties in the performance of everyday activities whether they are basic or more complex and are needed for living independently [6]. These basic and more complex tasks that are needed for independent living are often divided into (1) Activities of Daily Living (ADLs) which include personal basic activities such as hygiene and personal care, and (2) Instrumental Activities of Daily Living (IADLs) which include tasks that are needed to live in the community such as shopping, housekeeping, and meal preparation [7]. Both hearing and visual disability could significantly impact ADLs and IADLs. Visual disability, along with its impact in introducing difficulties when performing basic daily activities, is also associated with increased risk of falls, social isolation, and dependency [8]. Hearing disability also interferes with communication when listening and talking; adversely affecting socialization [9].

According to the World Health Organization, people with disabilities suffer poorer health outcomes [5]. One of the main reasons contributing to poor outcomes for this population is inadequate skillset of the healthcare providers [5]. In Saudi Arabia, The General Authority of Statistics has surveyed 25.13% of the disabled population in Riyadh city and added that 2.9% of the Saudi population have disabilities with an extreme effect on their daily lives [10]. The most prevalent disabilities by type include impaired mobility at 29%, followed by visual impairment at 24%, and the inability to communicate and understand at 10%, according to a disability survey conducted by the General Authority of Statistics in Saudi Arabia in 2017 [11]. This survey covers a random sample of 33,575 households, a nationally representative sample [11]. The sample was taken in light of results of the updated framework of the population and houses census of 2010 [11].

Patients with hearing or visual impairments encounter prehospital care difficulties in emergency situations such as difficulties in interacting with healthcare providers, communication issues, and lack of health and medical knowledge on which to rely when communication conditions are not ideal [5]. Despite these challenges, a retrospective cohort study by McKee and colleagues examined emergency department visits between hearing-impaired and non-hearing-impaired patients and reported that patients with impaired hearing are 1.94% more likely to utilize the emergency medical system than non-hearing-impaired patients [12]. Additionally, patients with vision and hearing loss encountered several difficulties when interacting with healthcare providers, including situations where they felt incompetent, unseen, or unheard [13].

Communication barriers have been a widespread issue affecting individuals with disabilities. Findings from recent evidence, which aimed to assess the perceptions of American Sign Language (ASL) interpreters about the barriers of communication between patients with hearing disability (deaf or hard of hearing) and healthcare professionals, showed that ASL interpreters observed in almost half of the appointments, patients with hearing disability did not understand the instructions given by healthcare professionals [14]. The ASL interpreters in that study also reported that healthcare professionals “hardly ever” use “teach-back” methods when communicating with these patients to ensure that they understand their instructions [14]. Indeed, 81% of the ASL interpreters reported that healthcare providers usually do not confirm if the patients understood any of the instructions by using any methods of confirmation [14]. The ASL interpreters also noted a lack of familiarization between the healthcare professionals and patients with hearing disability in relation to cultural and linguistic differences related to sign language and the deaf community, leading to ineffective forms of communication [14]. Given a variety of communication methods that may be used by patients with vision or hearing disabilities (e.g., text-reader devices, materials in braille, Structured Sign Language, drawing or writing, augmentative communication devices), and lack of appropriate tools and training to address these communication needs can cause clinical encounters to be ineffective and inefficient.

One of the reported issues when managing patients with hearing disability, as a result with communication issues, was underdiagnoses of diseases/conditions such as self-reported hypertension [15]. Such issues could result from communication barriers as healthcare providers had difficulties in explaining medical procedures and interventions, possibly affecting their outcomes [15]. Patients with disabilities also had difficulties reaching out and asking for prehospital care response. Mitigating protocols are already in order in Western developed countries and Australia to improve the access of patients with disabilities to healthcare systems including prehospital care [16, 17].

Broader literature showed that effective communication is correlated with better patients’ adherence to treatment [18]. Findings from a meta-analysis showed that patients whose physician is poorly communicating with them had 19% higher risk of non-adherence compared to those whose physician is communicating well [18]. The study also found that communication training for physicians could result in significant improvement of patients’ adherence to treatment, the odds of patients’ adherence was 1.62 times higher with physicians receiving communication training than those who have not received any training [18]. Another evidence showed that patients with preventable adverse events compared to those without were more likely to have communication barriers (language or disability) (odds ratio [OR] 3.00; 95% Confidence Interval [CI] 1.43–6.27) [19]. Main preventable adverse events included drug errors or poor clinical management [19]. The study, indeed, found that patients with communication barriers, in contrast with those without such barriers, were more likely to experience multiple preventable adverse events (46% v. 20%; p = 0.05) [19]. Furthermore, a recent study focusing on patients with hearing loss and patient satisfaction reported that patients with little trouble hearing and a lot of trouble hearing had higher odds of reporting dissatisfaction with care than those with no trouble hearing [(OR 1.47; 95% CI: 1.06, 2.03) and (OR 1.74; 95% CI: 1.15, 2.62), respectively] [20]. This could be attributed to communication barriers to hearing disability. Therefore, it is important to improve communication skills for healthcare professionals including paramedics to improve patient adherence to treatment and prevent adverse events that could be prevented through effective communication and improve patient satisfaction with care.

Patients with hearing disability are at risk of adverse outcomes. A recently published study from the United States of America reported that, compared to patients with good hearing, patients with little trouble of hearing were at increased risk of all-cause mortality (Hazard Ratio [HR] 1.17; 95% CI 1.13–1.20), a lot of trouble hearing (HR 1.45; 95% CI 1.40–1.50), and deaf (HR 1.54; 95% CI 1.38–1.73) [21]. The deaf category was found to have the highest risk of all-cause mortality and cause-specific cancer [21]. Findings from an earlier systematic review support these findings, showing a significant association between hearing disability and mortality after adjustment of all covariates [22]. The findings from the systematic review also showed that hearing disability was significantly associated with incident hospitalization (i.e., first hospitalization) and the number of hospitalizations per year [22]. Moreover, a recent study found that patients who had trouble communication due to hearing disability were more likely to be readmitted to hospital within 30 days (unadjusted OR 1.49; 95% CI 1.26–1.76; adjusted OR 1.32; 95% CI 1.06–1.64) [23]. On average, patients with trouble communication had 32% greater odds of hospital readmission in contrast with those with no hearing disability [23].

Research concerning the patients’ experiences, perceptions, and needs were extremely limited, in reviewing evidence among the visually impaired community. Similarly, the medical literature concerning prehospital care shortcomings and areas of possible growth in relation to those patients was also minimal. Multiple aspects of visual disability such as impaired contrast sensitivity and stereoacuity were shown to be significantly associated with walking limitations, potentially resulting in reduced mobility and ability to perform daily activities [24]. A recent systematic review of systematic reviews showed a consistent association between visual disability and reduced quality of life, highlighting the need to optimize healthcare for this population to improve their quality of life [25].

Overall, patients with hearing and/or visual disability are at risk of adverse outcomes including mortality, hospitalization, readmission, lower satisfaction with care, and reduced quality of life. There is a need to improve healthcare provided for this population including that of prehospital care to minimize the risk of such adverse outcomes and improve patients’ satisfaction with care. This includes, but not limited to, improving communication between healthcare professionals and patients with hearing/visual disability. This study, therefore, aimed to assess paramedics’ knowledge and attitude when managing patients with hearing or visual impairments. Moreover, it is intended to direct future research towards the identification of strengths and weaknesses in the paramedics’ knowledge and confidence when dealing with said patients.

## Methods

### Study design

A descriptive cross-sectional design was used to report the knowledge and experience of paramedics towards patients with hearing and/or visual disabilities in Saudi Arabia. A validated questionnaire was distributed to our study sample in Riyadh, Saudi Arabia between 01, July 2020 and 31, December 2020. Ethical approval was obtained from King Abdullah International Medical Research Center (approval number: SP20/108/R) in 09, June 2020.

### Setting

Data was collected from the Saudi Red Crescent Authority (SRCA) which is the main ambulance service in Saudi Arabia and emergency medical service departments in several hospitals and medical cities in Riyadh city, Saudi Arabia such as the Ministry of National Guard Health Affairs (MNGHA) and King Saud Medical City (KSMC) (see Supplement File 1).

### Survey administration and content

An online English self-administered questionnaire was distributed with consent form. The participants were asked to sign the consent before completing the questionnaire. Demographic data were collected with close ended questions. The survey included questions assessing knowledge, experience, and confidence in dealing with patients exhibiting visual and hearing impairments of the participating paramedics.

With literature paucity and unavailability of validated questionnaire that intended to measure the study outcome a questionnaire was developed for the study. The questionnaire development process took into consideration the study aim and targeted population. To maintain objectivity and consistency of the answer, a close ended questions and 5-point Likert scale was selected for the questionnaire. The questionnaire pilot assessment targeted randomly selected licensed paramedics and research experts working in King Saud bin Abdulaziz University for health sciences and King Abdulaziz Medical City at MNGHA, Riyadh, Saudi Arabia (3 researchers and 6 paramedics). A face validity method was followed to approve the survey before dissemination. The research team sent the survey to the invited researchers and paramedics and then, met with them online to take their feedback about the survey. The main aim was to assess the question flow, clarity, and logical reasoning of the questions to support the study aim. The pilot process revealed no major changes were required. The questionnaire was kept general and nonspecific to provide a snapshot of paramedics’ perspectives and did not include specifics such as the sign and symptoms of hearing loss, or methods of screening for hearing or vision issues with a hope of examining this in future research. The questionnaire contained sections for demographic information, a section to assess the paramedic’s experience in dealing with disability in the form of close ended questions, a section on knowledge assessment using close ended questions, and the paramedic’s level of confidence in managing patients with visual and hearing impairments using 5-point Likert Scale (see Supplement File 1).

The current study focused on hearing and visual disability /impairment rather than referring to only those diagnosed as deaf or blind. We elected to refer to disability as it imposes a more general condition where individual ability to communicate, interact, and move is limited.

For the purpose of this study, we inquired about the level of confidence and experience when handling patients with hearing and/or visual disabilities which limited their ability to effectively interact or communicate. We also inquired about paramedics’ experiences in patient encounters with hearing or vision disabilities throughout their careers. However, we recognize that in emergency situation with no previous given medical data, it is difficult to relay on the patient response or confusion as it may be the result of new underlying causes rather than a pre-existing hearing and visual disability.

It is important to highlight that the sign language the study referred to is unstructured sign language where paramedics use the sign language to point out on the affected body system rather than the structured Arabic alphabetic oriented sign language. We do agree on the importance of having training on structured language, however, not all patients and paramedics are educated on Arabic alphabetic oriented sign language and translators may not be readily available for encounters in emergency medical services.

### Participants and recruitment

In this study, the targeted populations were prehospital care providers working in Riyadh city, Saudi Arabia. The prehospital care system is predominantly run by paramedics and emergency medical technicians. Therefore, emergency doctors and emergency nurses were excluded. Based on paramedic license registration, there were approximately 1,500 paramedics in Riyadh city. With an alpha level of 0.05 and 95% confidence level. The approximate number of total paramedics working in Riyadh city was determined by contacting main ambulance service stakeholders and asking about the number of paramedics working at each ambulance service. The required sample size, calculated using Raosoft, was 310 participants. This means that we need exactly 310 returned questionnaires to be analysed to be statistically significant. To achieve this number of returned questionnaires, we increased our sample size by 20% (n = 372) as we expected a return rate of above 80% (around 83%) to achieve 310 returned responses. Although we knew that there were approximately 1,500 paramedics in Riyadh city, we do not know exactly how many of them are registered or not registered paramedics. Furthermore, the coronavirus disease 2019 (COVID-19) pandemic impacted the number of paramedics participation in any other tasks than clinical patient care due to high demand, but we did not know to what extent did this affect the number of paramedics working in the field. Therefore, a non-probability convenience sampling technique seems appropriate to apply in this study. All respondents in our survey are registered paramedics in Saudi Arabia. An online invitation was sent to the estimated 372 participants to complete the survey through Google Forms hyperlink. A reminder was sent to the participants to complete the survey. It was made clear that the participation in completing the survey is voluntary and anonymous as no personal identifiable information was collected. In addition, an informed consent form was completed before going ahead and completing the survey. Incomplete surveys were predetermined to be excluded from the study.

### Data analysis

All data was coded and entered in an Excel database. Data was analyzed using IBM SPSS statistics version 28 (IBM, New York, US). Descriptive data was presented as mean, median, and standard deviation for numerical data. For categorical data, percentage and frequency were used. The prevalence was estimated by recording the number of encounters divided by the total study population. To examine the relationship between the study variables and paramedic’s confidence level, the chi-square test was used for binary variables while Logistic Regression was used for multivariate variables. The confidence level based on 5-point Likert scale was divided into a binary outcome; 4 to 5 was considered confident and 3 or below was considered not confident. A p-value of 0.05 was considered as statistically significant.

## Results

### I: Descriptive statistics

We sent our survey to a total of 372 potential participants with the expectation that at least 310 questionnaires will be returned (83% assumed return rate). However, only 97 out of 372 participants completed the questionnaire, resulting in actual return rate of 26%. All surveys were complete and, therefore, no participants were excluded in this study. The majority of the participants were younger than 30 years (85%), were men (77%), and employed by Saudi Red Crescent Authority (25%) (Table 1).

### II: Participants experience in managing patients with hearing or visual impairment

**Fig. 1:**
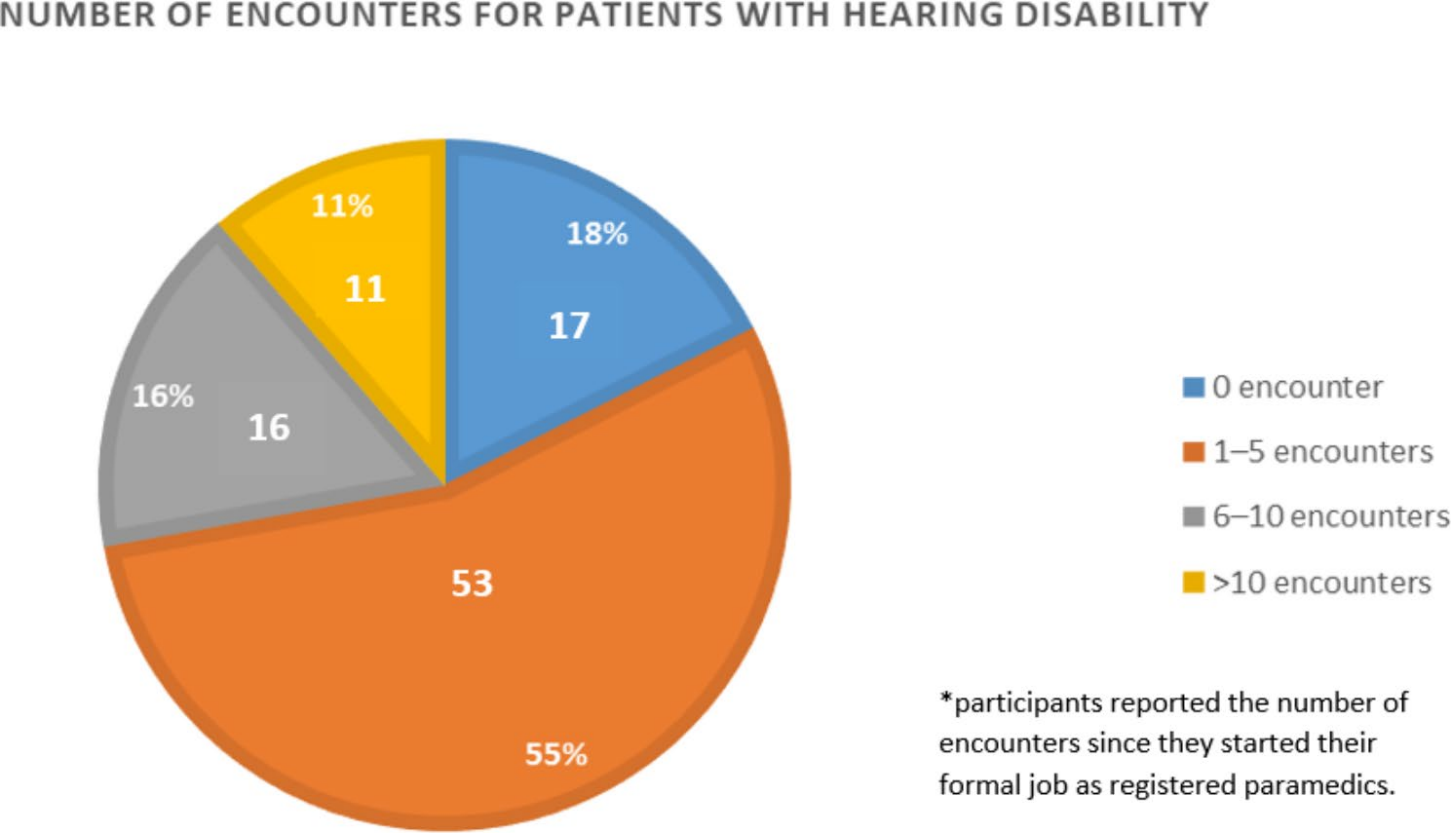
Paramedics’ encounters with patients suffering from hearing disability

**Fig. 2:**
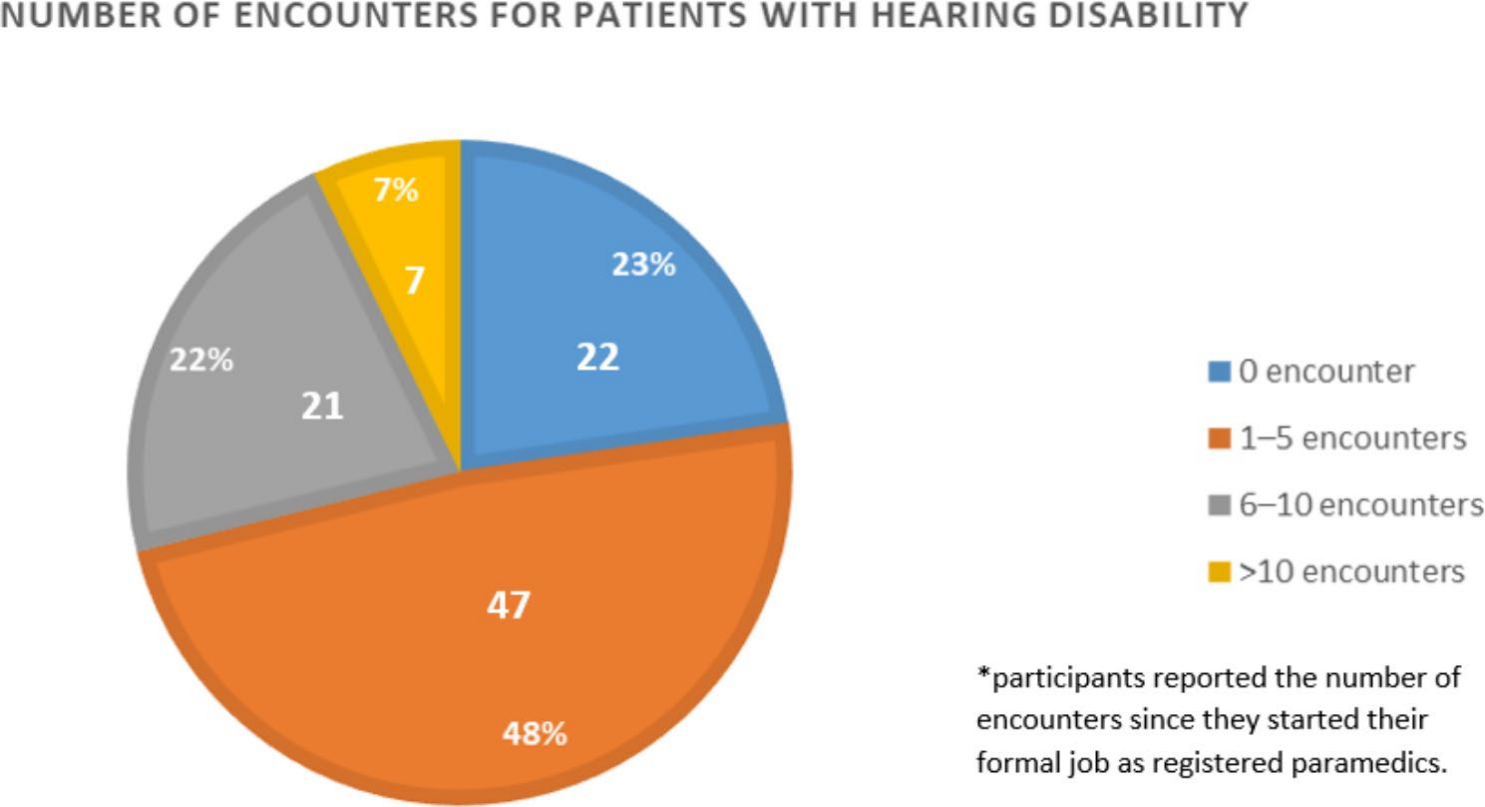
Paramedics’ encounters with patients suffering from visual disability

Of the 97 participants, 70 (72%) had encountered patients with visual and hearing impairments. Over the course of their careers, most participants encountered 1–5 cases for patients with hearing disability (55%) as well as patients with visual disability (48%) (Figs. 1 and 2).

Obtaining and documenting medical history was indicated by 42% of the participants as the biggest challenge when dealing with patients who had hearing or visual disability. Giving instructions and procedure explanation were the next two major difficulties as highlighted by 21% and 30% of the participants, respectively.

Regarding the tools that were used by the participants as means of communication, 35% of the participants used unstructured sign language and 14% used drawing. Alternatively, 33% of the participants indicated that they communicate with patients’ relatives on behalf of the patients. Participants indicated that around 70% of time history obtained from patient him/ herself, and around 80% from patient relatives (history was obtained in many cases from both patients and family members (not either or)). (Table 1)

Almost 50% of the participants indicated that they had not received any structured academic curriculum that is focused on managing patients with either hearing or visual disability. For skills training, 60% of the participants indicated that they had not received skills training to manage patients with hearing or visual disability. However, 85% of the participants showed an interest to receive structured education and training for managing these patients.

**Table 1:** Demographic characteristics of study variables

Variable	Number (%)
**Sex**	
Men Women	75 (77) 22 (23)
**Participant Occupation**	
Ministry of National Guard Health Affairs Saudi Red Crescent King Saud Medical City Others	18 (19) 24 (25) 4 (4) 51 (52)
**Age**	
20–25 26–30 31–35 > 35	55 (57) 28 (29) 12 (12) 2 (2)
**Place of Graduation**	
Governmental Saudi university Private Saudi university Overseas university	49 (50.5) 32 (33) 16 (16.5)
**Paramedics’ level of confidence**	
Not confident Confident	54 (56) 43 (44)
**Medical history obtained by patient**	
Yes No	75 (77) 22 (23)
**Medical history obtained by relatives**	
Yes No	80 (83) 17 (18)
**Means of communication with patients with hearing or visual disability**	
Unstructured sign language Relative (parents, siblings, and offspring) Writing Body language Drawing	34 (35) 32 (33) 26 (27) 16 (17) 14 (14)

### III: Paramedic’s confidence level in managing patients with visual and hearing disability

**Fig. 3:**
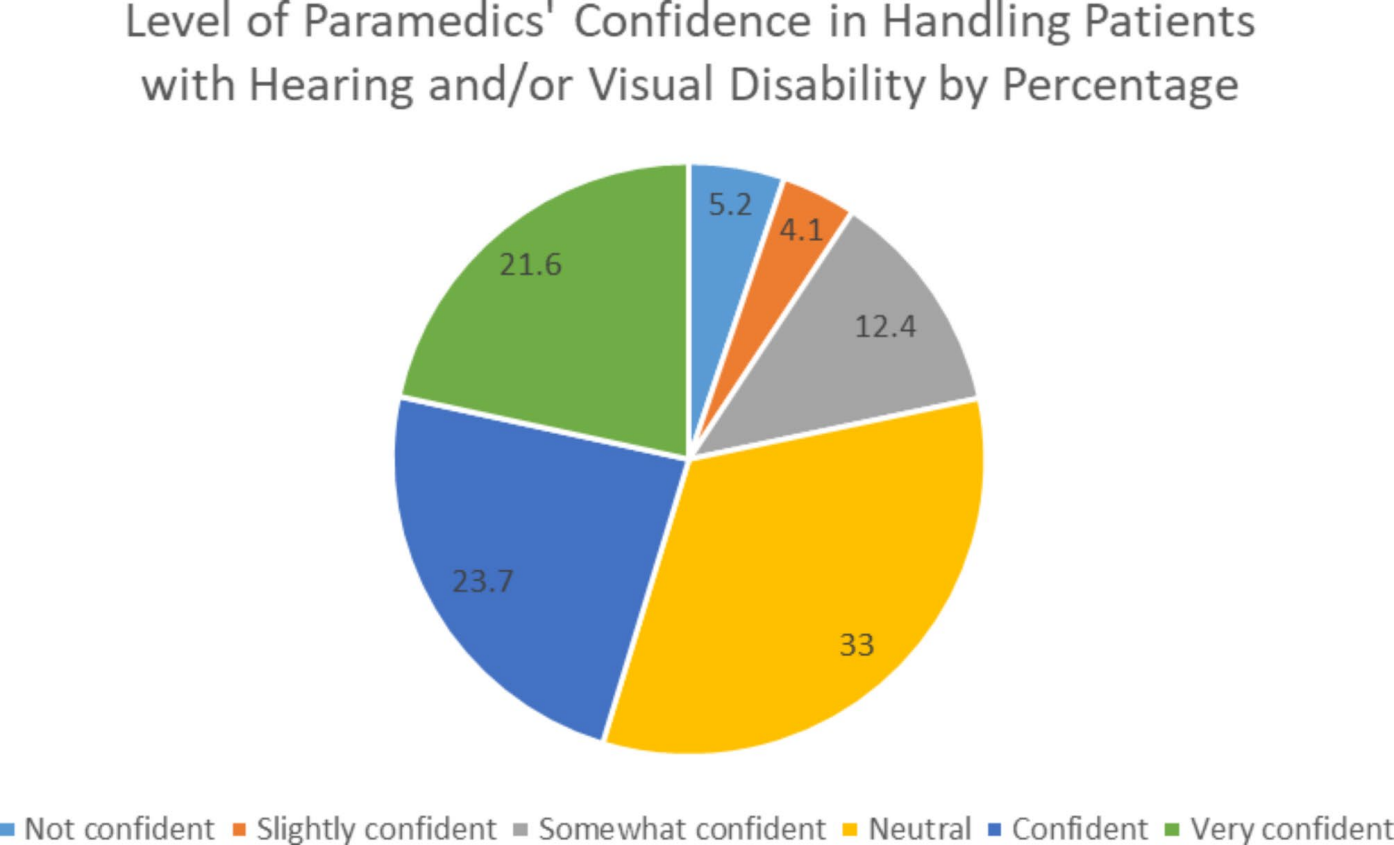
Participants’ level of confidence in managing patients with hearing and visual impairments

The majority of the participants reported a high level of confidence in managing patients with either hearing or visual disability (Fig. 3). Participants who had prior encounter with patients who had hearing disability were significantly more confident than those who had no previous experience (p-value = 0.039). However, the level of confidence was not statistically significant in the participants with previous experience in handling visually impaired patients (p-value = 0.279). Participants who obtained a structured academic curriculum were seven times more confident than participants who had no training (p-value = 0.006). This also applies to participants who obtained skill training; they were four times more confident than those who were not skilled (p-value = 0.03).

Moreover, the association between the study baseline information of the participants in the study and their level of confidence was assessed using logistic regression (Table 2). The findings showed no significant association between age, sex, place of graduation, and place of occupation with level of confidence (Table 2).

**Table 2:** Association between participant confidence level and study variables

Variable	Odds Ratio	95% Confidence interval	p-value
Age	1.119	0.768 − 1.799	0.457
Sex	1.016	0.363 - 2.450	0.904
Place of graduation	0.65	0.888 - 2.644	0.126
Place of occupation	0.49	0.721–1.385	0.999

## Discussion

To our knowledge, this is the first study to be published assessing the confidence and experience of paramedics when managing patients with hearing or visual disabilities in Saudi Arabia and one of the few studies internationally. The findings of this study showed that the majority (73%) of the study participants had encountered patients with visual and hearing impairments. Most of the participants encountered 1–5 cases patients with hearing or visual disability throughout their careers (49% and 55%, respectively). The biggest challenge reported when communicating with patients with hearing or visual disability was obtaining and documenting medical history followed by explaining procedures and giving instructions. Despite that it was deduced from the survey that the majority of times, medical history was obtained directly from the patient him/herself, and from the patients’ relatives at other times the participants reported high level of confidence with managing patients with hearing or visual disability. Prior encounter with patients with hearing disability only, obtaining a structured academic curriculum, and obtaining skill training to manage these patients were significantly associated with high level of confidence. Overall, the findings of this study showed interesting results which could significantly contribute to improving prehospital or emergency medical service care for this population.

The findings of our study showed that paramedics have communication barriers which could prevent them from obtaining medical history, explaining procedures and giving instructions. These findings are consistent with another study which showed that other healthcare providers had communication difficulties with patients with disabilities; adversely impacting their ongoing health care [26]. However, there is a paucity of evidence examining the communication difficulties for these patients in prehospital care.

One of the findings of this study highlighted that the level of confidence was not impacted by the participants’ age, place of occupation, nor place of graduation. Compared to a similar study concerned with exploring paramedics’ knowledge and experience when dealing with patients with communication disabilities, our findings support their conclusion that most of paramedics lacked confidence in their abilities regarding patients with disabilities despite their level of experience [27].

With regards to ways used by paramedics to communicate with patients with disabilities, unstructured sign language was the most used tool (35%) followed by drawings (14%) of the total means of communication. However, a recent study assessing the use of communication board by paramedics in prehospital care as an alternative means of communication did not mention any of the means of communication in our study to be used by paramedics [28]. In that study, paramedics felt more confident when using the board with 72% agreement that using the board aided the communication with the patients [28]. Another study emphasized the lack of utilizing such tools in current practice [29]. It also highlighted that healthcare providers when communicating with patients with hearing disabilities rarely used teach-back methods such as asking the patient to repeat what they heard in their own words [14]. Healthcare providers reported difficulties in giving instruction and in explaining procedures, which is mirrored in a different study that examined persons with disabilities as an unrecognized health disparity population [29]. The study noted that as these individuals with disabilities are not uniformly recognized as a health disparity population, this has been reflected in demonstrated inequitable access to medical communication accommodations and related academic curricula for health professionals [29].

It is clear that targeted education and training is needed, with an explicit need expressed from participating paramedics in the present study who wished to learn more on the topic. While examples of training in this specific context are few, other groups have developed broad-based disability training programs for practitioners that could be adapted for use among those with vision and hearing disabilities. One such study developed a training program for paramedics in emergency care for pediatric patients with special needs which presented in-depth knowledge concerning common complications that interfered with assessment and management for this population in emergency care [30]. The program included a manual and video with practice mannequins and skill evaluations. The findings from this study showed that paramedics who completed the program reported were more comfortable assessing and managing children with special health care needs [30], highlighting the need for training paramedics to appropriately assess and manage patients with disabilities.

### Strengths and limitations

The limitations of the study were related to the small sample size which may limit the generalizability of study findings. The 26% response rate was lower than anticipated. Furthermore, the study invited participants from Riyadh city only. This, therefore, could impact the generalizability of our study findings.

Another limitation is that our study did not address the kind of training that paramedics received pre-professionally or as continued professional education to assess and manage patients with hearing/visual disability. It only asked if paramedics had received academic or training courses specifically for this population. Our study also assessed the level of confidence based on what paramedics think of themselves when they assess and manage patients with hearing/visual disability. It did not include patients, so it is difficult to assume that paramedics who received academic or course training communicated well with these patients. Therefore, receiving such training should not be misconstrued as resulting in better services. Without the patient perspective, it is unknown whether their confidence that they communicated well is accurate.

Although we stated that this study focuses on hearing and visual disability rather than referring to only those diagnosed as deaf or blind and clearly defined this to the participants as mentioned in the methods section, we should highlight that some participants may have inadvertently underestimated the number of their patients with hearing or visual disability, as this is indicative of an issue that continues in health care and among the general population because hearing and visual loss are often gradual and subtle and health care workforce education on managing these complex cases are few. Moreover, in emergency situation with no previous given medical data, it is difficult to relay on the patient response or confusion as it may be the result of new underlying causes rather than a pre-existing hearing and visual disability. This could explain the reason that most of the participants in this study did not have training to communicate and manage patients with hearing/visual disability.

Recall bias also represents a limitation of the study as the study survey asked about the number of cases encountered and the training courses attended, which may introduce this type of bias.

However, the strength of this study is that it is the first, to our knowledge, to assess knowledge and experience of paramedics concerning patients with hearing and visual disability in Saudi Arabia and one of the few studies internationally. We believe that the findings of our study, although we have small sample size, could significantly aid current practice by providing better understanding the issues and available solutions when caring for patients with disabilities in prehospital care as well as direct future research in this aspect. The findings of this study could be also used to improve the awareness and provide targeted training among healthcare providers about their knowledge and level of confidence when caring for patients with disabilities. One strong suit of this study is the use of self-administered surveys so as to not influence the answers of the participants, which helped us conclude significant findings that otherwise might have been overlooked. Future large-scale national research is needed to explore and assess the use of formal training of paramedics on effective and appropriate care for patients with disabilities. Future studies could also investigate the use of communication boards and in prehospital care, which was not covered in the current study.

## Conclusion

This study showed that paramedics reported difficulties when communicating with patients with hearing or visual disability. They reported that they were not fully confident to care for these patients, suggesting the need for more education and training. The major difficulty when communicating with patients with hearing or visual disability was obtaining and documenting medical history followed by explaining procedures and giving instructions, both crucial to a successful health encounter. Most of the paramedics had encountered 1–5 cases of patients with such disabilities throughout their careers. We believe that the findings of this study are valuable for both future research and clinical practice. Further, training in effective communication for those with vision or hearing loss could improve health outcomes and satisfaction with care. Future research is needed to explore and assess the need for and use of formal training of paramedics on effective and appropriate care for patients with disabilities.

### Electronic supplementary material

Below is the link to the electronic supplementary material.

Supplementary Material 1

## Data Availability

The datasets used and/or analysed during the current study are available from the corresponding author on reasonable request.
