# Supplementary material for: Knowledge and experience of paramedics concerning patients with hearing and visual disability
Source: BMC Emerg Med. 2023 Aug 17;23:91. doi: 10.1186/s12873-023-00866-y (PMC10433550; doi:10.1186/s12873-023-00866-y)
Supplement: Supplementary file 1 — Supplementary Material 1 [file 12873_2023_866_MOESM1_ESM.docx]

Supplementary table 1 Study Questionnaire.

| **Personal Data** | |
| --- | --- |
| **Gender** | - Male - Female |
| **Age** | - 25 -30 - 31-35 - 36-40 - 41-45 - >45 years |
| **Years of experience** | - <2 years - 2-3 years - 3-4 years - >4 years |
| **Place of occupation** | - Saudi Red Crescent Authority. - Ministry of National Guard Health Affairs. - King Faisal Specialist Hospital. - King Saud Medical City. - King Abdullah bin Abdulaziz University Hospital. |
| **Where did you graduated from?** | Answer: |
| **Assessing the level experience in dealing with patients with hearing/visual disability** | |
| **Have you ever handled either adult or paediatric patients with hearing or visual impairments?** | - Yes - No |
| **Are you confident in handling patients with visual impairment?** | - Yes - No |
| **Are you confident in handling patients with hearing impairments?** | - Yes - No |
| **Estimate how many of said patients had a visual impairment?** | - 0 - 1-5 - 6-10 - >10 |
| **Estimate how many of said patients had a hearing impairment?** | - 0 - 1-5 - 6-10 - >10 |
| **Estimate the number of times history was taken directly from the patients themselves:** | - 0 - 1-5 - 6-10 - >10 |
| **Estimate the number of times history was taken from the relatives of the patient:** | - 0 - 1-5 - 6-10 - >10 |
| **Report the main difficulties you faced when dealing with those patients:** | - Taking history - Performing physical exam - Explaining procedure - Providing diagnosis - Giving instructions |
| **Report the tools /methods used to support you to communicate with patients:** | - Sign language - Drawing - Chart - Writing - Body language - Photo - Relative - Other, please mention: |
| **Measure level of confidence when dealing with visual or hearing impairment:** | - 1= no confidence - 2= slight confidence - 3= neutral - 4= moderate confidence - 5= high confidence |
| **Assessing the level of knowledge** | |
| **Number of academic courses taken concerning those patients:** | - 0 - 1-3 - 4-6 - >6 |
| **Number of training courses taken to aid in the care of patients with visual or hearing impairments:** | - 0 - 1-3 - 4-6 - >6 |
| **Do you have an interest in taking an academic/ training course regarding this subject:** | - Yes - No |
